# Photon number statistics uncover the fluctuations in non-equilibrium lattice dynamics

**DOI:** 10.1038/ncomms10249

**Published:** 2015-12-22

**Authors:** Martina Esposito, Kelvin Titimbo, Klaus Zimmermann, Francesca Giusti, Francesco Randi, Davide Boschetto, Fulvio Parmigiani, Roberto Floreanini, Fabio Benatti, Daniele Fausti

**Affiliations:** 1 Dipartimento di Fisica, Università di Trieste, Via Valerio 2 Trieste 34127, Italy; 2 Istituto Nazionale di Fisica Nucleare, Sezione di Trieste, Trieste 34014, Italy; 3 Ecole polytechnique, ENSTA ParisTech, CNRS, Université Paris-Saclay, 828 boulevard des Maréchaux, Palaiseau 91762, France; 4 Sincrotrone Trieste S.C.p.A., Basovizza 34127, Italy

## Abstract

Fluctuations of the atomic positions are at the core of a large class of unusual material properties ranging from quantum para-electricity to high temperature superconductivity. Their measurement in solids is the subject of an intense scientific debate focused on seeking a methodology capable of establishing a direct link between the variance of the atomic displacements and experimentally measurable observables. Here we address this issue by means of non-equilibrium optical experiments performed in shot-noise-limited regime. The variance of the time-dependent atomic positions and momenta is directly mapped into the quantum fluctuations of the photon number of the scattered probing light. A fully quantum description of the non-linear interaction between photonic and phononic fields is benchmarked by unveiling the squeezing of thermal phonons in α-quartz.

## Abstract (web summary)

Fluctuations of atomic positions are related to several materials properties. Here the authors measure the photon number statistics via a non-equilibrium optical experiment and provide a quantum description of the interaction between photonic and phononic fields to reveal lattice dynamics fluctuations in quartz.

## Graphical abstract

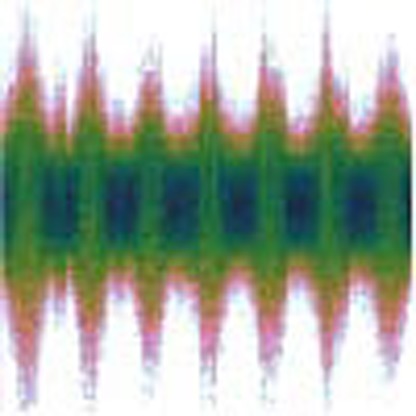

In a classical description, the displacement of the atoms along the vibrational eigenmodes of a crystal can be measured with unlimited precision. Conversely, in the quantum formalism positions and momenta of the atoms can be determined simultaneously only within the boundary given by the Heisenberg uncertainty principle. For this reason, in real materials, in addition to the thermal disorder, the atomic displacements are subject to fluctuations which are intrinsic to their quantum nature. While various evidences suggest that such quantum fluctuations may be of relevance in determining the onset of intriguing material properties, such as quantum para-electricity, charge density waves or even high temperature superconductivity^1^,^2^,^3^,^4^,^5^,^6^,^7^, the possibility of measuring quantum fluctuations in solids is the subject of an intense debate^8^,^9^,^10^,^11^,^12^,^13^,^14^,^15^,^16^,^17^,^18^,^19^,^20^,^21^.

The time evolution of atomic positions in materials is usually addressed by means of non-equilibrium optical spectroscopy. An ultrashort light pulse (the pump) impulsively perturbs the lattice and a second one (the probe), properly delayed in time, measures a response that is proportional to the spatially averaged instantaneous atomic positions. In those experiments, the time-dependent atomic displacements are often revealed by an oscillating response, commonly dubbed coherent phonon response^22^,^23^,^24^,^25^,^26^,^27^,^28^,^29^,^30^,^31^,^32^, at frequencies characteristic of the vibrational modes of the material. In this framework, it has been shown that a non-linear light–matter interaction can prepare non-classical vibrational states^8^,^13^ such as squeezed states, where the fluctuations of the lattice position (or momentum) can fall below the thermal limit. A reduction below the vacuum limit is known as vacuum squeezing^16^.

Here we propose a joint experimental and theoretical approach to access the fluctuations of the atomic positions in time domain studies. An experimental apparatus that allows for the measurement of the photon number quantum fluctuations of the scattered probe pulses in a pump and probe set-up is adopted. The connection between the measured photon number uncertainty and the fluctuations of the atomic positions is given by a fully quantum mechanical theoretical description of the time domain process. Overall we prove that, under appropriate experimental conditions, the fluctuations of the lattice displacements can be directly linked to the photon number quantum fluctuations of the scattered probe pulses. Our methodology, that combines non-linear spectroscopic techniques with a quantum description of the electromagnetic fields, is benchmarked on the measurement of phonon squeezing in α-quartz.

## Results

### Shot-noise-limited pump and probe experiments

In the non-linear spectroscopy formalism, the excitation mechanism of phonon states in transparent materials is called impulsive stimulated Raman scattering (ISRS)^29^. The susceptibility tensor *χ*^(3)^ connects the induced third order polarization *P*^(3)^ to three fields: 
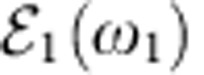
, 
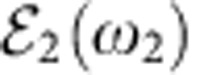
 and 
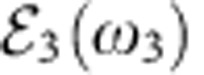
33. In conventional two pulses pump and probe experiments, the fields 
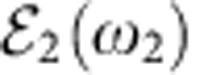
 and 
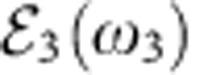
 are two different frequency components of the pump laser pulse. In particular, all photon pairs such that *ω*_3_−*ω*_2_=Ω, where Ω is the frequency of the Raman active vibrational mode, contribute to ISRS^34^. The interaction of the probe field 
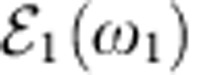
 with the photo-excited material induces an emitted field, 
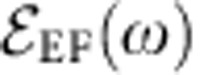
, which depends on the pump–probe delay and carries information about the specific Raman mode excited in the crystal. Experimental details are reported in (Supplementary Note 1). We choose a polarization layout designed to excite *E*-symmetry Raman optical modes in α-quartz at room temperature and get an emitted field with polarization orthogonal to the probe one^35^ (Supplementary Fig. 1).

**Figure 1:**
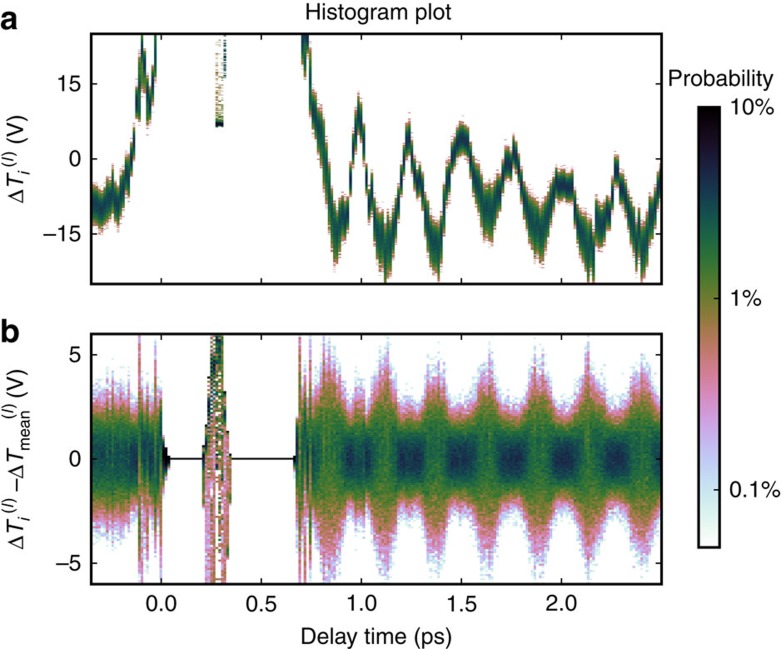
Time domain transmittance histogram plot. Δ*T*_*i*_ distribution as a function of pump–probe delay for a representative scan *l*. (**a**) For each time delay a colour plot of the normalized histogram of *N*=4,000 acquired pulses is shown. (**b**) Histogram plot of Δ*T*_*i*_ centred at zero. The data shown are obtained with the largest pump fluence used in the experiments (25 mJ cm^−2^).

The experimental layout is similar to standard pump and probe experiments. The sample is excited by an ultrashort pump pulse and the time evolution of the response is measured by means of a second much weaker probe pulse, which interacts with the photo-excited material at a delay time *τ*. The unique characteristics of our set-up are: unlike standard experiments, where the response is integrated over many repeated measurements, our system can measure individual pulses; the apparatus operates in low noise conditions allowing for the measurement of intrinsic photon number quantum fluctuations. In detail, we adopt a differential acquisition scheme where each probe pulse is referenced with a second pulse which has not interacted with the sample. For each measurement, the differential voltage is digitized and integrated, giving the transmittance Δ*T*_*i*_ for the *i*th measurement. For every given pump and probe delay *τ*, we repeat this single-pulse measurements for *N*=4,000 consecutive pulses. Figure 1a gives a useful visual representation of the obtained data. For one pump and probe scan *l*, the normalized histogram of *N*=4,000 acquired pulses for each delay time is shown. Each histogram represents the distribution of the measured Δ*T*_*i*_ for a specific delay time *τ*. For a clearer visualization of the physically meaningful information in the time evolution of the statistical distribution, Fig. 1b reports the histogram centred at zero.

The pump and probe scan is repeated several times and each *l*th scan provides 
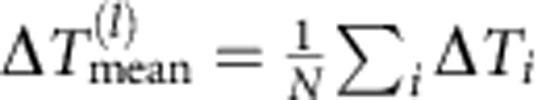
, and 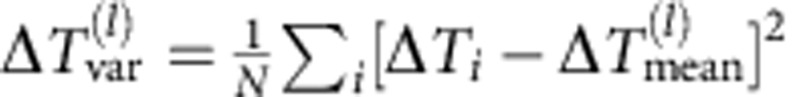. Finally the averages of these two quantities are calculated over all *M* scans as 
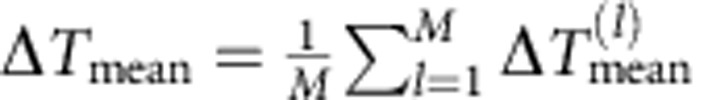
 and 
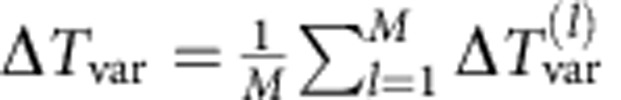
.

**Figure 2:**
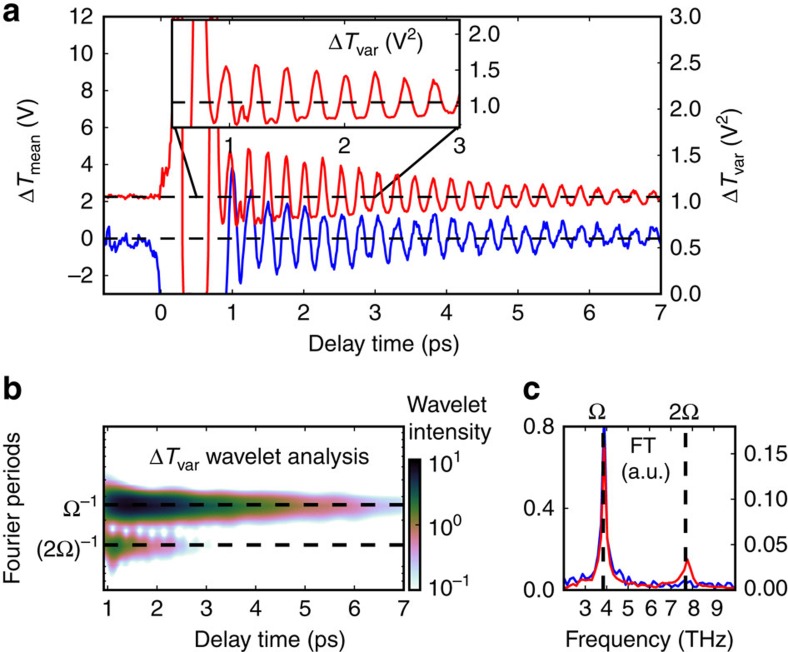
Time domain transmittance mean and variance. (**a**) Δ*T*_mean_ (blue curve) and Δ*T*_var_ (red curve) as a function of the pump–probe time delay. The zero time is the instant in which pump and probe arrive simultaneously on the sample. In the inset a zoom of the variance for the first 3 ps is shown. (**b**) Wavelet analysis (Morlet power spectrum) of the variance oscillating part. (**c**) Fourier transforms of the oscillating parts of mean (blue curve) and variance (red curve). In **a** and **c**, the left axis is related to the mean while the right axis is related to the variance.

The time domain response, averaged over *M*=10 scans, is shown in Fig. 2a for a representative pump fluence of 14 mJ cm^−2^ (a pump fluence-dependent study is reported later). The blue curve depicts the time evolution of the mean value of the transmittance Δ*T*_mean_, whereas the red curve shows the time evolution of its variance Δ*T*_var_. The Fourier transform of the mean (Fig. 2c, blue curve) has a single peak which is ascribed to the E-symmetry quartz vibrational mode with frequency Ω=128 cm^−1^=3.84 THz^36^. The same frequency component is observed in the Fourier transform of the variance (Fig. 2c, red curve). In addition, a second peak at twice the phonon frequency appears exclusively in the variance. A wavelet analysis of the variance oscillations allows for a time domain study of the two frequency components (Fig. 2b): one notices that while the fundamental frequency survives for roughly 7 ps, the 2Ω component vanishes within the first 2 ps. The different lifetimes between the Ω and 2Ω components of the variance are seen also by a close inspection of the raw data distribution plotted in Fig. 2b.

Note that the 2Ω in our data is visible only in experimental conditions where the noise is dominated by the quantum uncertainty, a situation which is known as shot-noise regime. In such conditions, Δ*T*_var_ measures the quantum variance of the scattered probe photon number. A full characterization of the detection system is reported in (Supplementary Note 2), including the shot-noise characterization (Supplementary Fig. 2) and the analysis of classical noise sources (Supplementary Figs 3 and 4). It should further be stressed that in experimental conditions where the noise is larger and dominated by classical sources, the 2Ω contribution to the noise becomes unmeasurable.

The presence of the 2Ω frequency component is suggestive of phonon squeezing, as it has been indicated by Raman tensor models^8^,^9^,^12^. Nevertheless, the experimental evidences up to date lack a direct comparison with a reliable quantum noise reference^10^,^11^,^13^,^20^. Hence, in these experiments the observation of the 2Ω frequency in the optical noise is considered as an indication of phonon squeezing, but not an unequivocal proof. In details, a 2Ω oscillating optical noise was reported in ref. 13, but later ascribed to an artifact^15^ due to the experimental amplification process. In particular, it has been demonstrated that amplification artifacts become more relevant when, using a lock-in amplifier-based acquisition, the time constant of the lock-in increases with respect to the time between steps in the pump–probe delay. This gives rise to maxima in the noise where the derivative of the mean signal is maximal^15^. Here we use a pump power density, which is almost 3 orders of magnitude higher than in refs 13, 15. In addition, we observe a 2Ω frequency component in the optical variance which exhibits maxima in correspondence with the minima of the derivative of the mean signal, hence ruling out possible artifacts of the kind described in (ref. 15).

### Fully quantum description of ISRS

To predict how the fluctuations of the atomic positions in a lattice can be mapped onto the photon number quantum fluctuations of the probe field, we develop a theoretical approach to time domain studies, which treats quantum mechanically both the material and the optical fields involved in the non-linear processes. Several semiclassical models describe the possibility of generating ‘classical' (coherent states) and non-classical vibrational states by photo-excitation. In particular, for transparent materials like quartz, the most commonly used approach is to adopt Raman tensor models where the interaction between photons and phonons is not mediated by dipole-allowed electronic transitions. In this condition, interactions linear in the phonon operators allow for the generation of coherent vibrational states, while high order interactions are required for the generation of non-classical squeezed states^9^,^12^,^32^. In materials with allowed dipole transitions, as in presence of excitons, different models based on electron–phonon coupling Hamiltonians have been proposed. In those models it has been shown that squeezed phonon states can result only by successive excitations with a pair of pulses^16^,^17^. All these models mainly adopt semiclassical approaches where the optical fields are described classically^33^, and therefore are unable to reproduce the quantum proprieties of the probe optical field that can be measured with the shot-noise-limited pump and probe set-up presented here. The key aspect of our approach, allowing us to bridge this gap, is to study both generation and detection of phonon states using a fully quantum formalism through an effective photon–phonon interaction, which is descriptive of experiments in transparent systems, such as α-quartz. The basic tool is a quantum Hamiltonian able to describe both pump and probe processes. Being linear and bilinear in the photon and phonon operators, this Hamiltonian accounts for the possible generation of coherent and squeezed phonon states through the pump process. In particular, it models also the detection of the photo-excited phonon states, describing the probing process by a fully quantum approach, providing in this way a direct comparison with the experimentally measured photon number quantum fluctuations of the scattered probe pulses^37^.

In this framework, the first step is to adopt a quantized description for the mode-locked pulsed laser fields^38^. Each mode of frequency *ω*_*j*_=*ω*_0_+*jδ*, where *ω*_0_ is the pulse central frequency, *δ* is a constant depending on the laser repetition rate and *j* is an integer, is quantized and described by single-mode creation and annihilation operators 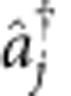 and 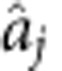. In this framework, ISRS can be modelled by means of an effective impulsive interaction Hamiltonian, which is descriptive of both the pumping and the probing processes. In both processes, two optical fields with orthogonal polarizations (denoted with subscript *x* or *y*) are involved: two pump fields in the pumping process and the probe and the emitted field in the probing process. The interaction Hamiltonian has the form


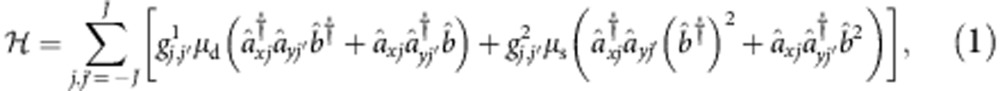


where 2*J*+1 is the total number of modes within a mode-locked optical pulse, 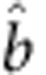 and 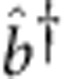 are the phonon annihilation and creation operators; *μ*_d_ and *μ*_s_ are coupling constants and the function 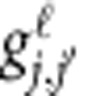 takes into account the relations between the frequencies of the involved fields,


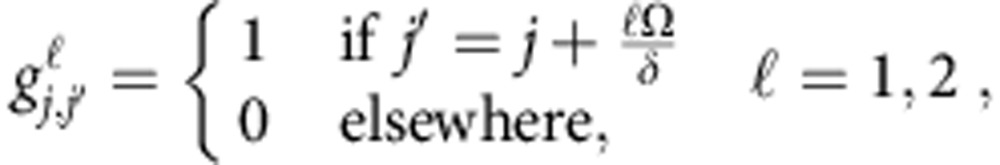


with Ω the phonon frequency. A complete interaction Hamiltonian should contain also second order processes involving phonons with opposite momenta. However, since the probe detects only the **k**≃0 optical transition, we can make use of an effective Hamiltonian that accounts only for this kind of process.

**Figure 3:**
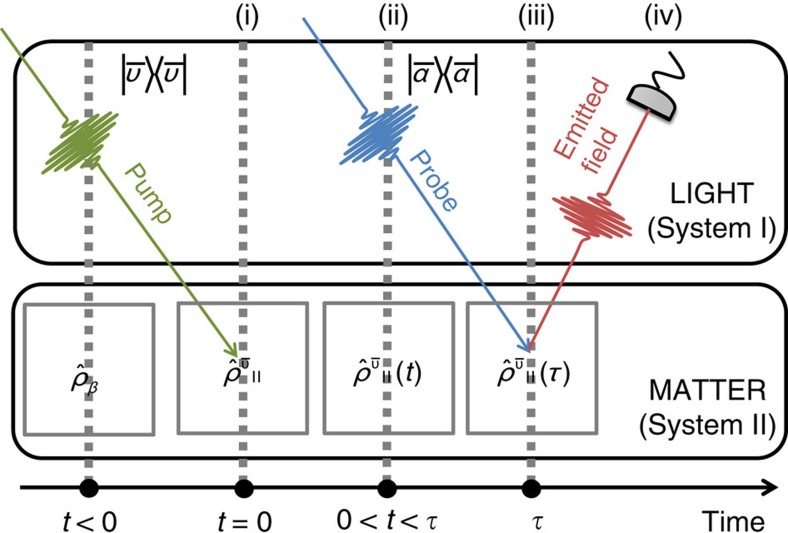
Sketch of the four-step effective theoretical model. The steps are indicated with roman numbers. The details of the theory for each step are reported in the text. The photon and phonon system are denoted with I and II, respectively.

The whole theoretical description of the experiment can be rationalized in a four-step process as sketched in Fig. 3: (i) generation of phonon states in the pumping process, (ii) evolution of the produced vibrational state, (iii) probing process and (iv) read out of the emitted photon observables.

(i) Initially, the sample is in thermal equilibrium and it is described by a thermal phonon state 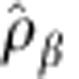, at inverse temperature *β*. The laser pump pulse is described by a multimode coherent state of high intensity 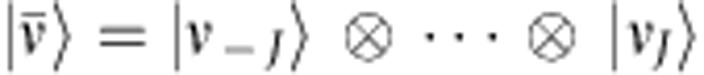, where 
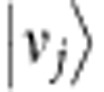
 are single-mode coherent states associated with all the frequency components within the pulse. Each 
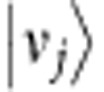
 is an eigenstate of the annihilation operator 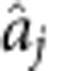 of photons in the mode of frequency *ω*_*j*_, 
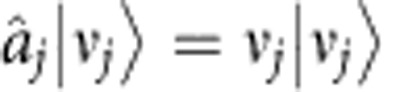
. We indicate with 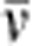 the vector whose components are the amplitudes *ν*_*j*_. The equilibrium (pre-pump) photon–phonon state 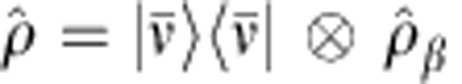 is instantaneously transformed into 
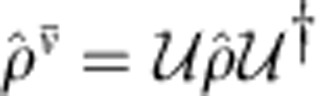
 by means of the unitary operator 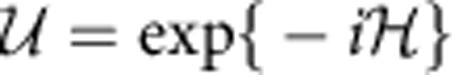. Since the pumping operator 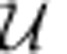 acts on a high intensity photon coherent state 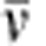, we can use the mean field approximation for the photon degrees of freedom and replace 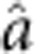 with *ν* and 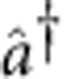 with ν* for both pump modes involved in equation (1), thus replacing 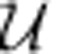 by


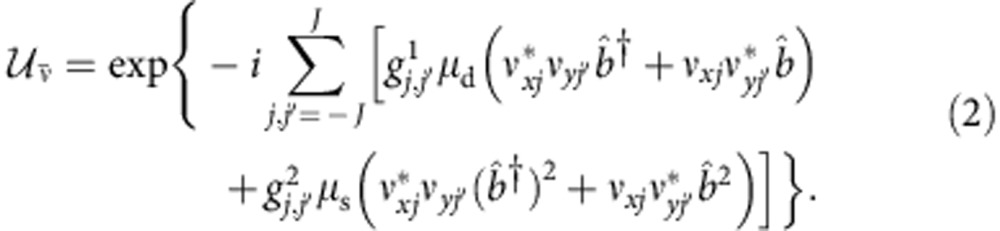


The evolution operator generates coherent and squeezed phonon states, respectively, through the linear and quadratic terms in the phonon operators 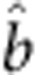 and 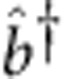. The initial state 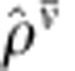 contains information about both photons and phonons. Tracing over the photon degrees of freedom, the resulting state 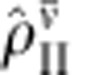 describes the excited phonons brought out of equilibrium by the impulsive pump process.

(ii) The time evolution of the excited phonons is described by using an open quantum systems approach, namely by means of a suitable master equation of Lindblad form^39^,^40^ that takes into account, besides the quantum unitary evolution, also the dissipative and noisy effects due to the interaction with a thermal environment.

(iii) The incoming probe pulses are in the multimode coherent state 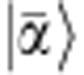. The probing process at time *τ* is described by the same impulsive unitary operator 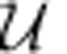 used for the pump. However, in this case we can apply the mean field approximation only to the probe photon operators with *x* polarization, which correspond to a much more intense field than those with *y* polarization. Moreover, since the probe field is much weaker than the pump field, the quadratic terms in the interaction Hamiltonian in equation (1) can now be neglected. The resulting unitary operator is


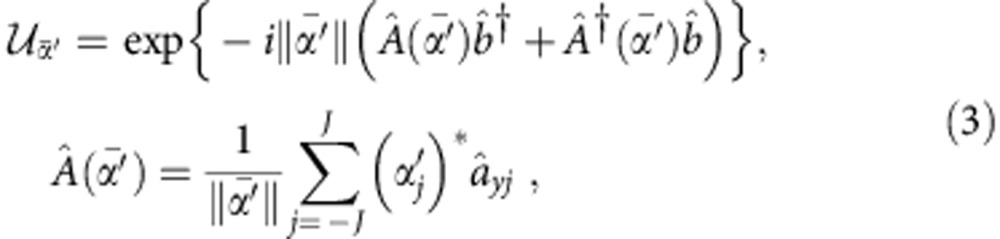


where 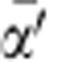 is the vector with components 
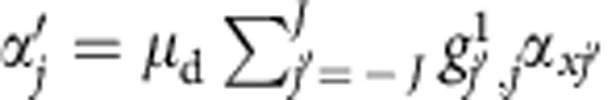
 and 
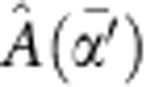
 is a collective photon annihilation operator such that 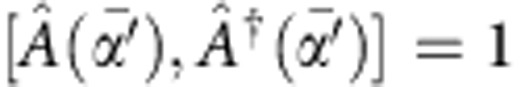.

**Figure 4:**
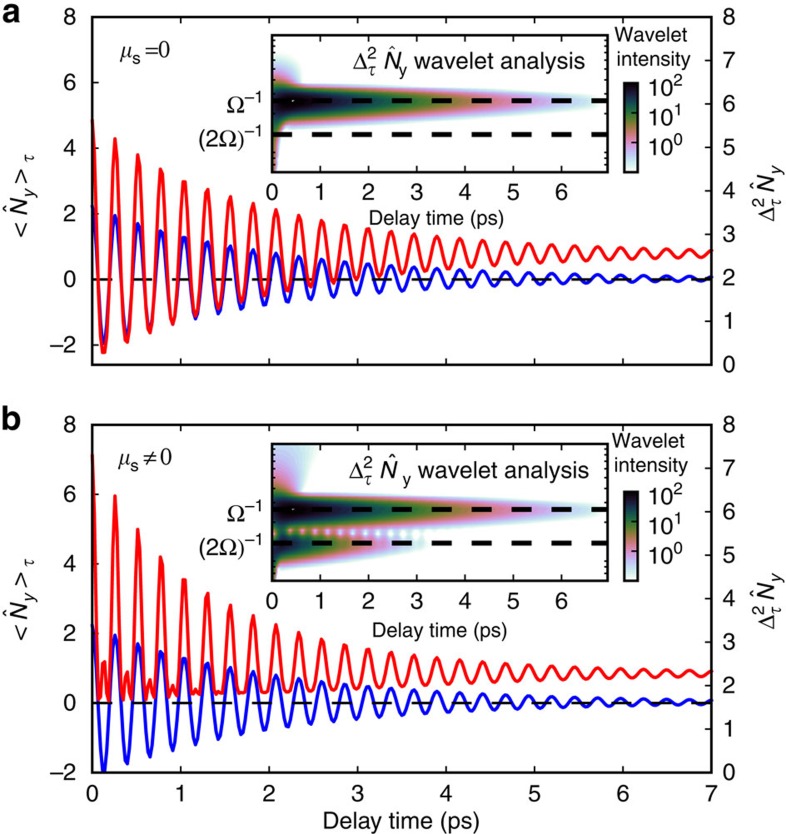
Model predictions. Theoretical calculations of the mean value and the variance of the number of photons of the emitted field. The left axis is related to the mean while the right axis is related to the variance. Comparison between the case with squeezing coupling constant *μ*_s_=0 (**a**) and *μ*_s_≠0 (**b**). A wavelet analysis (Morlet power spectrum) of the variance is reported in the inset for both cases.

The latter unitary operator acts on a state of the form 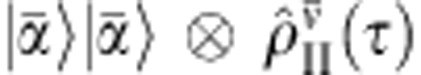. The information about the phonons are extracted by measuring the emitted field photons. In particular, the emitted photon state 
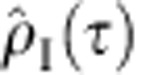
 is obtained by tracing away the phonon degrees of freedom.

(iv) The possible quantum features of the phonon state, for example, squeezing, can be read off as they are imprinted into 
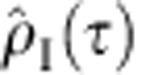
. In particular for each time delay *τ*, we can compute the quantities 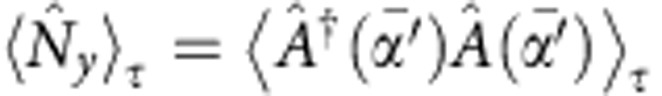 and 
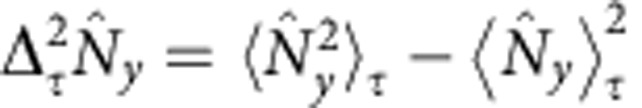
, which correspond to the observables measured in the experiment that are the mean value and the variance of the number of photons of the emitted field. The details of the theoretical model are reported in (Supplementary Note 3). The theoretical results for *μ*_s_=0 and *μ*_s_≠0 are shown in Fig. 4 together with the corresponding wavelet analysis for the variance of the number of emitted photons. The calculations reproduce the experimental results in Fig. 2, revealing a 2Ω frequency component in the variance, only when the pump creates squeezed phonon states (*μ*_s_≠0). In particular, for *μ*_s_≠0, the model reproduces the different lifetimes between the Ω and 2Ω components in the variance observed in the experiments. The explicit expressions for the theoretically predicted amplitudes of both the frequency components in the variance are reported in (Supplementary Note 3), showing that the same damping constant, characterizing the dissipative phonon time evolution, contributes differently to the two frequency components giving rise to different decay times.

## Discussion

**Figure 5:**
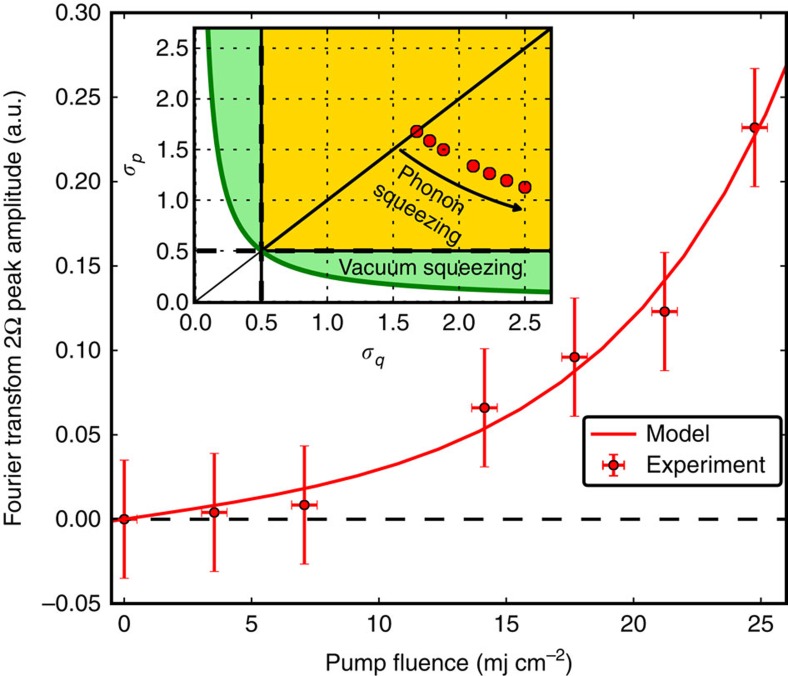
Fourier transform 2Ω peak amplitude of the variance. Amplitude of the 2Ω peak of the Fourier transform of the time-dependent variance Δ*T*_var_. The error bars indicate the s.d. over 10 scans. Comparison between experiments and theory as a function of the pump fluence. In the inset the uncertainties for the phonon position and momentum operators, calculated from the model, are plotted for the corresponding pump fluences.

The proposed effective interaction model is further validated by a pump fluence dependence study. Figure 5 shows the amplitude of the 2Ω peak in the Fourier transform of the variance, Δ*T*_var_, as a function of the pump fluence. A fluence dependence study of the Ω peak is reported in (Supplementary Fig. 5). The functional behaviour obtained from the model predictions (continuous line in Fig. 5) agrees with the experimental data only in presence of a pump-induced squeezing of the phonon mode (*μ*_s_≠0). The increase of the 2Ω peak amplitude with the pump fluence allows us to give a direct estimation of the uncertainties of the phonon-conjugated quadratures, which are reported in the inset of Fig. 5 for the different excitation fluences (calculation details are given in (Supplementary Note 3)). For high pump fluences, the uncertainty on one of the phonon quadratures falls below the thermal limit at the equilibrium, indicating the squeezed nature of the photo-excited thermal vibrational states. Our experimental approach allows for the direct measurement of the photon number quantum fluctuations of the probing light in the shot-noise regime and our fully quantum model for time domain experiments maps the phonon quantum fluctuations into such photon number quantum fluctuations, thereby providing an absolute reference for the vibrational quantum noise. The comparison of the predicted noise with the experimental photon number quantum uncertainty, measured in shot-noise conditions, allows us to unveil non-classical vibrational states produced by photo-excitation. A future extension of the model taking into account the role of the electronic degrees of freedom would allow to extend such a study from transparent materials to complex absorbing systems.

In conclusion, a Raman active phonon mode has been impulsively excited via ISRS in a α-quartz by means of a pump and probe transmittance experiment with single-pulse differential acquisition in noise conditions limited by intrinsic probe photon number fluctuations. A fully quantum mechanical effective model where both phonons generation and detection are studied through the same effective coupling Hamiltonian establishes a direct connection between the measured photon number quantum fluctuations of the emitted probe field and the fluctuations of the atomic positions in a real material. Our approach is used here to reveal distinctive quantum properties of vibrational states in matter, in particular the squeezed nature of photo-excited phonon states in α-quartz. Finally, we stress that our approach can be applied in future studies addressing the role of unconventional vibrational states in complex systems^3^,^6^, and the thermodynamics of vibrational states^41^,^42^ possibly in the quantum regime.

## Additional information

**How to cite this article:** Esposito, M. *et al.* Photon number statistics uncover the uctuations in non-equilibrium lattice dynamics. *Nat. Commun.* 6:10249 doi: 10.1038/ncomms10249 (2015).

## Supplementary Material

Supplementary Information Supplementary Figures 1-5, Supplementary Notes 1-3 and Supplementary References
