# Corrigendum: Photon number statistics uncover the fluctuations in non-equilibrium lattice dynamics

**DOI:** 10.1038/ncomms10651

**Published:** 2016-02-01

**Authors:** Martina Esposito, Kelvin Titimbo, Klaus Zimmermann, Francesca Giusti, Francesco Randi, Davide Boschetto, Fulvio Parmigiani, Roberto Floreanini, Fabio Benatti, Daniele Fausti

Nature Communications 6: Article number: 10249 10.1038/ncomms10249 (2015); Published: 12222015; Updated: 02012016

The affiliation details for Davide Boschetto are incorrect in this article. The correct affiliation details for this author are given below:

Laboratoire d'Optique Appliquée, ENSTA ParisTech, CNRS, Ecole polytechnique, Université Paris-Saclay, 828 boulevard des Maréchaux, 91762 Palaiseau cedex, France.

