# Supplementary material for: Photon number statistics uncover the fluctuations in non-equilibrium lattice dynamics
Source: Nat Commun. 2015 Dec 22;6:10249. doi: 10.1038/ncomms10249 (PMC4703887; doi:10.1038/ncomms10249)
Supplement: Supplementary Information — Supplementary Figures 1-5, Supplementary Notes 1-3 and Supplementary References [file ncomms10249-s1.pdf]

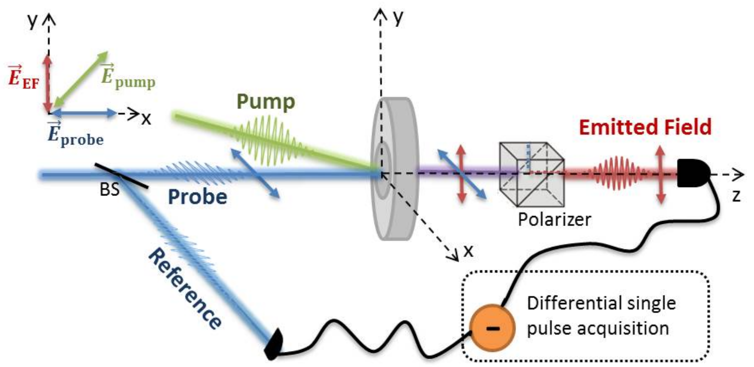


**Supplementary Figure** 1: **Scheme of the experimental setup.** The sample is depicted at the origin of the coordinates. The polarization configuration of pump, probe and emitted field is indicated on top-left. After the interaction with the sample the probe undergoes a polarization selection in order to detect the emitted field only. The differential acquisition system is also sketched.

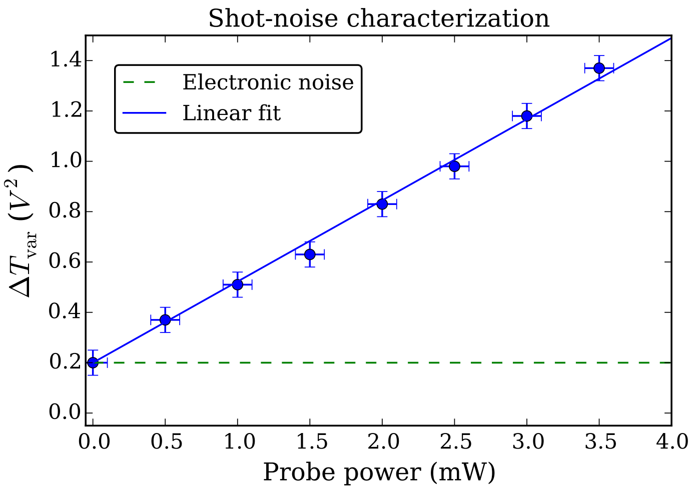


**Supplementary Figure** 2: **Shot-noise characterization of the detection apparatus**. Variance of 4000 acquired differential pulses as a function of the probe power. The vertical error bars indicate the standard deviation over 10 repeated measurements, the horizontal error bars indicate the instrumental error of the power-meter.

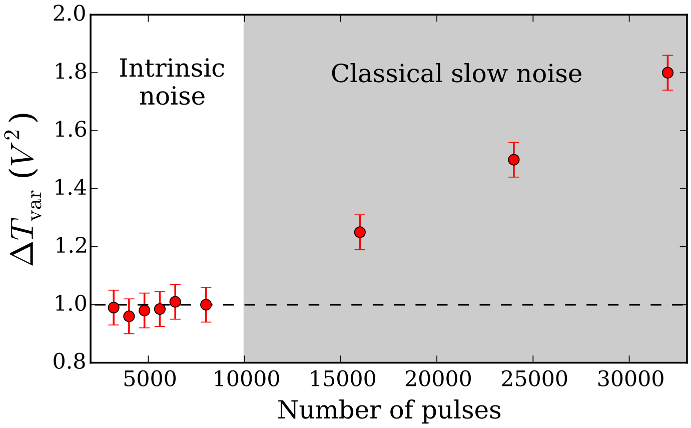


**Supplementary Figure** 3: **Noise characterization as a function of the number of acquired pulses.** Variance, $\Delta T_{\rm var}$, in absence of the pump, as a function of the number of acquired successive pulses. The dashed line indicates the shot-noise level. The grey area represents the region in which classical slow noise sources contributes to the variance. The error bars indicate the standard deviation over 10 repeated measurements.

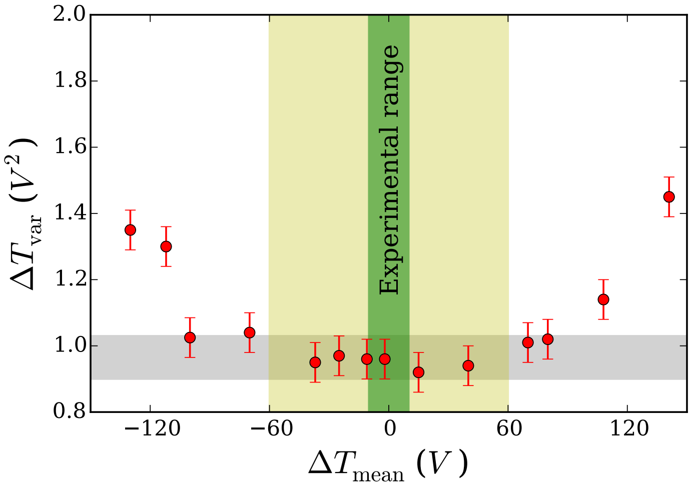


**Supplementary Figure** 4: **Noise characterization as a function of the unbalance.** Variance, $\Delta T_{\mathrm{var}}$, in absence of the pump for a fixed number of pulses ($N = 800$), as a function of the unbalance between the transmitted probe pulse and the reference pulse, expressed as acquired mean voltage $\Delta T_{\mathrm{mean}}$. Grey area: range of minimal noise; yellow area: unbalance region in which the noise randomly fluctuates in a range of minimal values; green area: unbalance region in which the experiments have been performed. Notice that we use the convention of expressing the voltage acquired for every single pulse acquisition as the sum of the voltages digitized for 500 points at 1 $\mathrm{GS\,s^{-1}}$. For reference, the digitized measurement of a differential pulse with a 1 V ($-1$ V) voltage peak corresponds to a value of 300 V ($-300$ V). The error bars indicate the standard deviation over 10 repeated measurements.

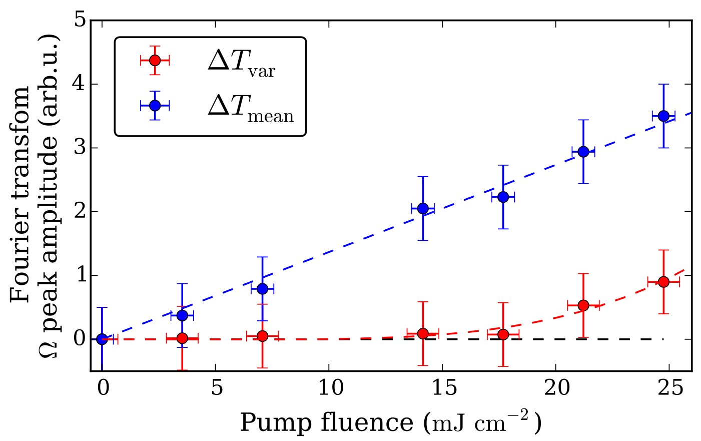


**Supplementary Figure** 5: **Amplitude of the Ω peak of the Fourier transform**. Fourier transform Ω peak of the time dependent mean, $\Delta T_{\mathrm{mean}}$, (blue points) and of the time dependent variance, $\Delta T_{\mathrm{var}}$, (red points). The dashed lines are a guide for the eyes. The error bars indicate the standard deviation over 10 scans.

## Supplementary Note 1. Polarization dependent ISRS in quartz

Impulsive stimulated Raman scattering (ISRS) is a non resonant excitation mechanism of lattice vibrations in transparent materials by ultrashort laser pulses. It is a four-wave mixing process due to third order polarization effects. The Cartesian components of the third order non-linear polarization are given by

$$P_i^{(3)}(\omega) = \sum_{jkl} \chi^{(3)}_{ijkl} \, [\mathcal{E}_1(\omega_1)]_j \, [\mathcal{E}_2(\omega_2)]_k \, [\mathcal{E}_3(\omega_3)]_l, \tag{1}$$

where $\chi^{(3)}_{ijkl}$ is the susceptibility tensor, $\mathcal{E}_1$ is the probe field and $\mathcal{E}_2$ and $\mathcal{E}_3$ are the pump fields. The susceptibility tensor determines the polarization selection of vibrational modes that can be excited via ISRS [1].
In particular, quartz Raman active vibrational modes are 4 totally symmetric modes of symmetry $A_1$ and 8 doubly degenerate modes of symmetry $E$ (transverse and longitudinal) [2].

In our experiment, the sample is a 1 mm thick $\alpha$-quartz, oriented in order to have the principal symmetry axis parallel to the probe propagation direction. A scheme of the chosen experimental geometry is shown in Supplementary Figure 1. Both pump and probe come from the same laser source, a 250 kHz mode-locked amplified Ti:Sapphire system. The pulse duration is 80 fs, the fractional change in the probe transmission due to the pump is of the order of 5% for a pump fluence of $25\,\mathrm{mJ\,cm^{-2}}$. The excited phonon state is detected via the scattering of the probe pulse which arrives on the sample with time delay $\tau$ with respect to the pump. The transmitted light undergoes a polarization selection through a polarizer positioned after the sample.

The pump direction is almost collinear with the probe one. Assuming that the involved optical fields propagate along the $z$ direction, we can limit our analysis to the $xy$ plane. In this case the quartz Raman tensors assume the form [3]

$$A = \begin{pmatrix} a & 0 \\ 0 & a \end{pmatrix} \quad E^T = \begin{pmatrix} c & 0 \\ 0 & -c \end{pmatrix} \quad E^L = \begin{pmatrix} 0 & -c \\ -c & 0 \end{pmatrix}. \tag{2}$$

Following the notation in [4], the susceptibility tensor can be expressed as:

$$\chi^{(3)}_{ijkl} = A_{ij}\, A_{kl} + E^T_{ij}\, E^T_{kl} + E^L_{ij}\, E^L_{kl}, \tag{3}$$

where each index can assume the values $1, 2$ associated to the direction $x$ and $y$ respectively. Thus, the susceptibility tensor $\chi^{(3)}_{ijkl}$ gives rise to a $4 \times 4$ block-matrix

$$\begin{pmatrix} \begin{pmatrix} a^2+c^2 & 0 \\ 0 & a^2-c^2 \end{pmatrix} & \begin{pmatrix} 0 & c^2 \\ c^2 & 0 \end{pmatrix} \\ \\ \begin{pmatrix} 0 & c^2 \\ c^2 & 0 \end{pmatrix} & \begin{pmatrix} a^2-c^2 & 0 \\ 0 & a^2+c^2 \end{pmatrix} \end{pmatrix}. \tag{4}$$

The first two indexes refer to the outer matrix elements and describe the polarization components of the emitted field ($i$ index) and of the probe field ($j$ index), while the last two indexes ($k$ and $l$) indicate the inner matrix elements and describe the polarization components of the two pump fields.

In particular, we are interested in selecting the excitation of an $E$ symmetry Raman mode. For this purpose we use a probe linearly polarized along $x$ and we perform a polarization selection after the sample in order to detect the emitted field component orthogonal to the probe (along $y$). This polarization configuration allows the selection of the susceptibility matrix elements $\chi^{(3)}_{21kl}$ associated with the involved $E$ phononic mode. Notice that such elements vanish when $k = l$ that is when the two pump fields are both polarized along $x$ or along $y$. Thus, in order to activate the process, we need the two pump fields (two frequency components of the same laser pulse) to have orthogonal polarizations. This is possible when the pump pulse is linearly polarized along a direction in between $x$ and $y$. In particular, the efficiency of the ISRS is maximal when the pump polarization is at $45\,^\circ$ with respect to the $x$ axis. This is indeed the configuration we chose and consequently the matrix elements involved in our experiment are $\chi^{(3)}_{21kl(k\neq l)} = c^2$, getting an emitted field almost collinear with the unscattered probe photons and with polarization orthogonal to the probe one. We configure a polarizer after the sample in order to transmit the emitted field polarization only. The global polarization configuration is sketched in Supplementary Figure 1.

Note that a full extinction of the unscattered probe is experimentally not achievable (polarizer extinction rate $10^5$). The residual probe acts as a local oscillator amplifying the emitted field within the total signal [5].

## Supplementary Note 2. Single pulse differential acquisition system in shot-noise limited regime

The acquisition system is made of a balanced amplified differential photodetector and a fast digitizer (*Spectrum* M3i.2132-exp) with sampling rate 1 $\mathrm{GS\,s^{-1}}$. The differential photodetector consists of two *Hamamatsu S3883* Silicon PIN photodiodes with 0.94 quantum efficiency connected in reverse bias and followed by a low-noise charge amplifier. The photo-currents generated by the two photodiodes in response to a single optical pulse impinging on them (transmitted probe pulse on the first photodiode and reference pulse on the second photodiode) are physically subtracted and the resulting charge is amplified using *CAEN* custom designed electronic components. In particular, the preamplifier sensitivity is 5.2 $\mathrm{mV\,fC^{-1}}$ with a linear response up to about 2 V of pulse peak voltage. Note that we use here the convention of expressing the voltage acquired for every single differential pulse acquisition, $\Delta T_i$ , as the sum of the voltages digitized for 500 points at 1 $\mathrm{GS\,s^{-1}}$. For reference, the digitized measurement of a pulse with a 1 V voltage peak corresponds to a value of 300 V.

Notice that our acquisition apparatus distinguishes itself by avoiding the lock-in amplification and the possible artifacts associated with its use [6]: the single pulse differential acquisition system we adopted allows the individual measurement of each single transmitted probe pulse, and gives access to the intrinsic photon-number quantum fluctuations.

In order to distinguish the intrinsic noise (shot-noise) from other contributions, we tested the experimental set-up in absence of the pump. The variance $\Delta T_{\mathrm{var}}$ of 4000 differential pulses is measured for different powers of the probe. The optical noise, shown in Supplementary Figure 2, is linear with a constant offset representing the electronic noise. This behavior is characteristic of the shot-noise regime [7]. We chose a probe power within the linearity interval (2.5 mW, 0.2 $\mathrm{mJ\,cm^{-2}}$ on the sample; note that this corresponds roughly to $10^6$ photons per pulse scattered in cross polarization on the detector). This provides a reference for the shot noise value of about 1 $\mathrm{V}^2$ which is used to benchmark the noise in time domain experiments and avoid additional noise sources. The detector shot-noise linearity test demonstrates that we are sensible to quantum fluctuations of the photon number. In particular, the shot-to-electronic noise at the maximum probe intensity in the linear regime is approximately 10 dB.

We further characterized our experimental apparatus by measuring the variance, $\Delta T_{\mathrm{var}}$, in absence of the pump, as a function of the number of acquired pulses. The results are shown in Supplementary Figure 3. We observe that the variance increases when the number of acquired successive pulses increases, this means that for long acquisitions a slow noise contribution makes higher the measured variance (grey area in Supplementary Figure 3). Thus, we chose to acquire $N = 4000$ successive pulses per step in order to guarantee the statistical meaningfulness of the data but at the same time avoiding contributions of classical slow noises. Note that the time to time noise in the mean number of photons reported in the main text is larger than the value of the variance. This is explained by considering that the fluctuations in the mean values are made large by slow noise of classical nature (the differences between the measurements scan by scan). On the contrary the variance is dominated by intrinsic fluctuations and, as discussed in the main text, it is calculated for every scan separately and then averaged. The results reported in Supplementary Figure 3 show that acquiring up to about $10^4$ pulses guarantees that classical slow noise contributions are excluded.

Moreover we measured the obtained variance $\Delta T_{\mathrm{var}}$ for a fixed number of pulses ($N = 800$) in absence of the pump as a function of the unbalance between the transmitted probe pulse and the reference pulse, that is as a function of the acquired mean voltage $\Delta T_{\mathrm{mean}}$. The results of such characterization measurement are shown in Supplementary Figure 4. One can notice that the noise randomly fluctuates in a range of minimal values (grey area) for small positive or negative unbalance (yellow area). For larger unbalance the noise starts to increase due to artifacts in the amplification process. All the time domain experiments reported here have been performed within the region of small detector unbalancing (green area) in order to be sure of working in shot noise limited conditions.

The results of our time resolved experiments show the presence of a $2\Omega$ frequency component only in the variance of the probe photon number. A pump fluence dependent study of the of the amplitude of the $2\Omega$ peak in the Fourier transform of the variance is reported in the main text. For completeness we report here the amplitude of the $\Omega$ peak in the Fourier Transform of the mean, $\Delta T_{\mathrm{mean}}$, and of the variance, $\Delta T_{\mathrm{var}}$, as a function of the pump fluence. The data are show in Supplementary Figure 5.

## Supplementary Note 3. An effective fully quantum mechanical model for ISRS

The effective fully quantum mechanical approach to ISRS followed in the main text consists in the pump process, the subsequent dissipative, irreversible phonon dynamics and the probe process, all of them being described by quantum dynamical maps [8].

Before being hit by the pump laser beam described by photons in a multi-mode coherent state $|\bar{\nu}\rangle\langle\bar{\nu}|$, the state of the relevant phonon mode at frequency $\Omega$ is appropriately taken to be a thermal state at inverse temperature $\beta$

$$\hat{\rho}_\beta = \left(1 - \mathrm{e}^{-\beta\Omega}\right) \mathrm{e}^{-\beta\Omega\, \hat{b}^\dagger \hat{b}} \ . \tag{5}$$

The pump process is characterized by a photon-phonon interaction Hamiltonian of the form

$$\mathcal{H} = \sum_{j,j'=-J}^{J} \left[ g^1_{j,j'}\, \mu_\mathrm{d} \big(\hat{a}^\dagger_{xj}\, \hat{a}_{yj'}\, \hat{b}^\dagger + \hat{a}_{xj}\, \hat{a}^\dagger_{yj'}\, \hat{b}\big) + g^2_{j,j'}\, \mu_\mathrm{s} \big(\hat{a}^\dagger_{xj}\, \hat{a}_{yj'}\, (\hat{b}^\dagger)^2 + \hat{a}_{xj}\, \hat{a}^\dagger_{yj'}\, \hat{b}^2\big)\right] , \tag{6}$$

where $\mu_\mathrm{d}$ and $\mu_\mathrm{s}$ are coupling constants, $2J+1$ is the total number of modes within a mode-locked optical pulse, and the functions $g^\ell_{j,j'}$ take into account the relations between the frequencies of the involved fields,

$$g^\ell_{j,j'} = \begin{cases} 1 & \text{if } j' = j + \frac{\ell\Omega}{\delta} \\ 0 & \text{elsewhere,} \end{cases} \qquad \ell = 1, 2 \ .$$

As stated in the main text, it should be noted that while the linear term involves only the creation of a phonon in a single mode at null momentum $\mathbf{k}$, the quartic term are not limited to $\mathbf{k} = 0$ and one should integrate over the entire optical phonon dispersion including processes where the momentum conservation is guaranteed by the creation of optical phonons with opposite momenta [9, 10]. In our effective Hamiltonian we include only a single phonon mode at $\mathbf{k} = 0$. This assumption is made in view of the fact that, in the performed experiments, the probing process is limited to the linear regime so that phonons at $\mathbf{k} \neq 0$ will not affect the observed photon number fluctuations in this configuration.

Initially, the sample is in thermal equilibrium and it is described by a thermal phonon state $\hat{\rho}_\beta$, at inverse temperature $\beta$. The Hamiltonian in (6) generates an impulsive change of the initial photon-phonon state $|\bar{\nu}\rangle\langle\bar{\nu}| \otimes \hat{\rho}_\beta$ given by

$$\hat{\rho}^{\bar{\nu}} = \mathcal{U}\,(|\bar{\nu}\rangle\langle\bar{\nu}| \otimes \hat{\rho}_\beta)\,\mathcal{U}^\dagger = |\bar{\nu}\rangle\langle\bar{\nu}| \otimes \mathcal{U}_{\bar{\nu}}\, \hat{\rho}_\beta\, \mathcal{U}^\dagger_{\bar{\nu}} \ , \tag{7}$$

where, because of the high intensity of the pump laser beam, we have adopted the mean field approximation and substituted the photon annihilation and creation operators by the scalar amplitudes $\nu$ and $\nu^*$ and replaced $\mathcal{U}$ with

$$\mathcal{U}_{\bar{\nu}} = \exp\{-i[c_1\, \hat{b}^\dagger + c_1^*\, \hat{b} + c_2\, (\hat{b}^\dagger)^2 + c_2^*\, \hat{b}^2]\} \tag{8}$$

$$c_1 = \mu_\mathrm{d} \sum_{j,j'=-J}^{J} g^1_{j,j'}\, \nu^*_{xj}\, \nu_{yj'} \tag{9}$$

$$c_2 = \mu_\mathrm{s} \sum_{j,j'=-J}^{J} g^2_{j,j'}\, \nu^*_{xj}\, \nu_{yj'} \ . \tag{10}$$

The pump thus prepares the relevant phonon degree of freedom in a state $\hat{\rho}^{\bar{\nu}}_{\mathrm{II}}$ which is obtained from $\hat{\rho}^{\bar{\nu}}$ by tracing over the photon degrees of freedom:

$$\hat{\rho}^{\bar{\nu}}_{\mathrm{II}} = \mathrm{Tr}_{\mathrm{I}}(\,\hat{\rho}^{\bar{\nu}}) = \mathcal{U}_{\bar{\nu}}\, \hat{\rho}_\beta\, \mathcal{U}^\dagger_{\bar{\nu}} \ , \tag{11}$$

where II and I refer to the phonon and photon system, respectively.

The linear contribution in the phonon operators is responsible for the displacement of $\hat{b}$ and $\hat{b}^\dagger$, while the quadratic one accounts for their multiplication by hyperbolic functions and thus for the possible squeezing of the corresponding quadratures [11]:

$$\mathcal{U}^\dagger_{\bar{\nu}} \begin{pmatrix} \hat{b} \\ \hat{b}^\dagger \end{pmatrix} \mathcal{U}_{\bar{\nu}} = \mathbf{S} \begin{pmatrix} \hat{b} \\ \hat{b}^\dagger \end{pmatrix} + \frac{1}{2|c_2|^2} (\mathbf{S} - 1) \begin{pmatrix} c_1^* c_2 \\ c_1 c_2^* \end{pmatrix} \tag{12}$$

$$\mathbf{S} = \begin{pmatrix} \cosh(2|c_2|) & -\mathrm{e}^{i(\phi + \frac{\pi}{2})} \sinh(2|c_2|) \\ -\mathrm{e}^{-i(\phi + \frac{\pi}{2})} \sinh(2|c_2|) & \cosh(2|c_2|) \end{pmatrix} , \tag{13}$$

where $c_2 = |c_2|\mathrm{e}^{i\phi}$. In order to write the squeezing matrix $\mathbf{S}$ in the standard formalism [11], we can define for convenience a complex squeezing parameter

$$\xi = r\mathrm{e}^{i\psi}, \qquad \text{where} \qquad r = 2|c_2| = 2\,|\mu_\mathrm{s}| \sum_{j,j'=-J}^{J} g^2_{j,j'}\, \nu^*_{xj}\, \nu_{yj'}, \qquad \text{and} \qquad \psi = \phi + \frac{\pi}{2}. \tag{14}$$

Notice that the squeezing parameter amplitude $r$ depends linearly on the intensity of the pump pulse and on the squeezing coupling constant $\mu_{\rm s}$ which weights the non linear term in the interaction Hamiltonian and models the material properties involved in the process.

The variance of the quadrature operator $\hat{B} = \frac{\hat{b}+\hat{b}^\dagger}{\sqrt{2}}$ with respect to the state $\hat{\rho}_{\rm II}^{\bar{\nu}}$ is given by

$$\Delta^2_{\hat{\rho}_{\rm II}^{\bar{\nu}}}\hat{B} = {\rm Tr}_{\rm II}\left(\hat{\rho}_{\rm II}^{\bar{\nu}}\hat{B}^2\right) - \left({\rm Tr}_{\rm II}\left(\hat{\rho}_{\rm II}^{\bar{\nu}}\hat{B}\right)\right)^2 = \frac{1}{2}\coth\left(\frac{\beta\Omega}{2}\right)\left[\cosh(2r) - \sinh(2r)\cos\psi\right] \ . \tag{15}$$

Then, for $\psi = 0$ and $r$ large enough, one can make $\Delta^2_{\hat{\rho}_{\rm II}^{\bar{\nu}}}\hat{B}$ smaller than $1/2$ which is the shot noise variance of $\hat{B}$ with respect to the vacuum state $|0\rangle$ such that $\hat{b}|0\rangle = 0$.

The photoexcited phonon state $\hat{\rho}_{\rm II}^{\bar{\nu}}$ then undergoes a dissipative dynamics that effectively takes into account the interaction of the phonons with their environment until, after a delay time $\tau$, the target is hit by the probe laser beam. The phonon dynamics is considered to be that of an open quantum system in weak interaction with a large heat bath that will eventually drive the time-evolving phonon density matrix $\hat{\rho}_{\rm II}^{\bar{\nu}}(t) = \hat{\rho}_{\rm b}(t)$ to a thermal state $\hat{\rho}_{\beta'}$ at temperature $T'$ larger than that of the pre-pump phonon state: $\beta' \le \beta$. Such a relaxation process is described by a master equation [12, 13] for the phonon density matrix $\hat{\rho}_{\rm b}(t)$ of the form $\partial_t\hat{\rho}_{\rm b}(t) = \mathbb{L}[\hat{\rho}_{\rm b}(t)]$, where the generator of the time evolution is given by

$$\begin{aligned} \mathbb{L}[\hat{\rho}_{\rm b}(t)] &= -i\Big[\Omega\,\hat{b}^\dagger\hat{b}\,,\,\hat{\rho}_{\rm b}(t)\Big] \\ &+ \lambda\,(1+n')\left(\hat{b}\,\hat{\rho}_{\rm b}(t)\,\hat{b}^\dagger - \frac{1}{2}\Big\{\hat{b}^\dagger\hat{b}\,,\,\hat{\rho}_{\rm b}(t)\Big\}\right) \\ &+ \lambda\,n'\left(\hat{b}^\dagger\,\hat{\rho}_{\rm b}(t)\,\hat{b} - \frac{1}{2}\Big\{\hat{b}\hat{b}^\dagger\,,\,\hat{\rho}_{\rm b}(t)\Big\}\right) \ , \end{aligned} \tag{16}$$

where $n' = \dfrac{1}{{\rm e}^{\beta'\Omega}-1}$ $(> n = \frac{1}{{\rm e}^{\beta\Omega}-1})$, while $\lambda$ is a coupling constant sufficiently small so that the non-negligible presence of the environment can nonetheless be accounted for, in the so-called weak-coupling limit regime [12], by a master equation of the above type.

The first term of $\mathbb{L}$ generates the rotation in time of the phonon mode phase at its own eigenfrequency. The second two contributions consist of a so-called noise term $\hat{b}\,\hat{\rho}_{\rm b}(t)\,\hat{b}^\dagger$, respectively $\hat{b}^\dagger\,\hat{\rho}_{\rm b}(t)\,\hat{b}$ that has the property of transforming pure states into mixed states and of a dissipative term $-\frac{1}{2}\Big\{\hat{b}^\dagger\hat{b}\,,\,\hat{\rho}_{\rm b}(t)\Big\}$, respectively $-\frac{1}{2}\Big\{\hat{b}\hat{b}^\dagger\,,\,\hat{\rho}_{\rm b}(t)\Big\}$. These terms counterbalance the noise by keeping the trace of the time-evolving state $\hat{\rho}_{\rm b}(t)$, and thus the overall probability, constant in time. The anti-commutators can be incorporated into the Hamiltonian as anti-Hermitian contributions responsible for exponential time relaxation. The structure of $\mathbb{L}$ is such that the generated time-evolution maps, formally $\gamma_t = \exp(t\mathbb{L})$, compose as a forward-in-time semigroup: $\gamma_t \circ \gamma_s = \gamma_s \circ \gamma_t = \gamma_{t+s}$ for all $s, t \ge 0$. Moreover, $\hat{\rho}_{\rm b}(t) = \gamma_t[\hat{\rho}_{\rm b}]$ can be explicitly computed for any initial phonon state $\hat{\rho}_{\rm b}$; all initial states are eventually driven to a unique invariant state satisfying $\mathbb{L}[\hat{\rho}_{\rm b}] = 0$ that is given by the thermal state $\hat{\rho}_{\beta'}$.

Finally, the probe process is again described by the Hamiltonian in equation (6). However, the corresponding impulsive unitary operator $\mathcal{U} = \exp\left(-i\mathcal{H}\right)$ now acts on a photon-phonon state of the form $|\bar{\alpha}\rangle\,\langle\bar{\alpha}|\,\otimes\,\hat{\rho}_{\rm II}^{\bar{\nu}}(\tau)$. Here, $|\bar{\alpha}\rangle\,\langle\bar{\alpha}|$ is the multi-mode coherent state associated with the probe laser beam which contains $x$ and $y$ polarized components and is much less intense than the pump one, while $\hat{\rho}_{\rm II}^{\bar{\nu}}(\tau)$ is the phonon state dissipatively evolved up to the delay time $\tau$ between pump and probe. Differently from the pump process, the lower probe intensity allows one to neglect in $\mathcal{H}$ the quartic terms responsible for the squeezing effects. Moreover, we can apply the mean field approximation only to the field operators with $x$ polarization, since these probe components are much more intense than those polarized along $y$. Then, by replacing $\hat{a}_{xj}$ and $\hat{a}^\dagger_{xj}$ by $\alpha_{xj}$ and $\alpha^*_{xj}$ the probe process is described by

$$\mathcal{U}_{\bar{\alpha}'} = \exp\{-i\|\bar{\alpha}'\|\big(\hat{A}(\bar{\alpha}')\,\hat{b}^\dagger + \hat{A}^\dagger(\bar{\alpha}')\,\hat{b}\big)\} \ , \tag{17}$$

where $\hat{A}(\bar{\alpha}')$ is the collective photon annihilation operator

$$\hat{A}(\bar{\alpha}') = \frac{1}{\|\bar{\alpha}'\|}\sum_{j=-J}^{J}(\alpha'_j)^*\,\hat{a}_{yj} \ , \quad \alpha'_j = \mu_{\rm d}\sum_{j'=-J}^{J} g^1_{j',j}\,\alpha_{xj} \ . \tag{18}$$

Then, the probe process affects an initial state $|\bar{\alpha}_y\rangle\,\langle\bar{\alpha}_y|\,\otimes\,\hat{\rho}_{\rm II}^{\bar{\nu}}(\tau)$, where $|\bar{\alpha}_y\rangle = |\alpha_{y\,-J}\rangle\otimes\cdots\otimes|\alpha_{y\,J}\rangle$ is the coherent state involving only the $y$ polarization components such that $\hat{a}_{yj}\,|\bar{\alpha}_y\rangle = \alpha_{yj}\,|\bar{\alpha}_y\rangle$.
Notice that, unlike in (7), $\mathcal{U}_{\bar{\alpha}'}$ acts on the photon-phonon state as a whole and transforms it into

$$\mathcal{U}_{\bar{\alpha}'}\;|\bar{\alpha}_y\rangle\,\langle\bar{\alpha}_y|\,\otimes\,\hat{\rho}_{\rm II}^{\nu}(\tau)\mathcal{U}^\dagger_{\bar{\alpha}'} \ . \tag{19}$$

This allows for the quantum features of the phonon state and of its dynamics to be transcribed onto the emitted photon state

$$\hat{\rho}_{\rm I}(\tau) = {\rm Tr}_{\rm II}\left(\mathcal{U}_{\bar{\alpha}'}\;|\bar{\alpha}_y\rangle\,\langle\bar{\alpha}_y|\,\otimes\,\hat{\rho}_{\rm II}^{\nu}(\tau)\mathcal{U}^\dagger_{\bar{\alpha}'}\right) \ . \tag{20}$$

Unlike in the semi-classical theoretical approaches to pump and probe experiments attempted so far, one can here confront the experimental data not only with the scattered probe beam intensity, namely with the mean photon number $\langle\hat{N}_y\rangle_\tau$, where $\hat{N}_y = \hat{A}^\dagger(\bar{\alpha}')\hat{A}(\bar{\alpha}')$ and

$$\langle\hat{N}_y\rangle_\tau = \mathrm{Tr}\left(\hat{N}_y\,\hat{\rho}_{\mathrm{I}}(\tau)\right)\ , \tag{21}$$

but also with its variance $\Delta^2_\tau\hat{N}_y = \langle\hat{N}_y^2\rangle_\tau - \langle\hat{N}_y\rangle_\tau^2$.
Then one uses that

$$U^\dagger_{\bar{\alpha}'}\,\hat{N}_y\,U_{\bar{\alpha}'} \;=\; A^\dagger(\bar{\alpha}')\hat{A}(\bar{\alpha}')\cos^2(\|\bar{\alpha}'\|) + \hat{b}^\dagger\hat{b}\sin^2(\|\bar{\alpha}'\|) + \frac{i}{2}\sin(2\|\bar{\alpha}'\|)\big(A(\bar{\alpha}')\hat{b}^\dagger + A^\dagger(\bar{\alpha}')\hat{b}\big), \tag{22}$$

where, given the experimental conditions effectively described by the model, it is plausible to set all amplitudes $\alpha_{xj} = \alpha_x$ and $\alpha_{yj} = \alpha_y = |\alpha_y|\exp(i\theta_y)$, in which case

$$\alpha'_j = \mu_{\mathrm{d}}\alpha_x = |\mu_{\mathrm{d}}\alpha_x|e^{i\theta'}, \qquad \text{and} \qquad \|\bar{\alpha}'\| = \sqrt{K}|\mu_{\mathrm{d}}\alpha_x|\,, \tag{23}$$

where $K = 2J+1$ is the total number of modes within a mode-locked optical pulse.
By denoting with $I_y = K|\alpha_y|^2$ the pulse intensity for the $y$ polarization and using that

$$\hat{A}(\bar{\alpha}')\,|\bar{\alpha}_y\rangle = \sqrt{I_y}e^{-i(\theta'-\theta_y)}\,|\bar{\alpha}_y\rangle\,, \tag{24}$$

one explicitly computes:

$$\langle\hat{N}_y\rangle_\tau \;=\; I_y\cos^2(\|\bar{\alpha}'\|) + \sin^2(\|\bar{\alpha}'\|)\,\langle\hat{b}^\dagger\hat{b}\rangle_\tau + \frac{i}{2}\sqrt{I_y}\,\sin(2\|\bar{\alpha}'\|)\left(e^{-i(\theta'-\theta_y)}\,\langle\hat{b}^\dagger\rangle_\tau - e^{i(\theta'-\theta_y)}\,\langle\hat{b}\rangle_\tau\right), \tag{25}$$

where $\langle\hat{O}\rangle_\tau = \mathrm{Tr}_{\mathrm{II}}\left(\hat{\rho}^{\bar{\nu}}_{\mathrm{II}}(\tau)\,\hat{O}\right)$ is the expectation value of any phonon operator $\hat{O}$ with respect to the phonon state $\hat{\rho}^{\bar{\nu}}_{\mathrm{II}}(\tau)$.
Despite its complicated expression, we report also the number variance $\Delta^2_\tau\hat{N}_y$ predicted by the model, as $\langle\hat{N}_y\rangle_\tau$ and $\Delta^2_\tau\hat{N}_y$ are the quantities computed numerically in the main text and compared with the experimental data:

$$\begin{aligned}
\Delta^2_\tau\hat{N}_y \;=\;& I_y\cos^4(\|\bar{\alpha}'\|) + \sin^4(\|\bar{\alpha}'\|)\left(\langle(\hat{b}^\dagger\hat{b})^2\rangle_\tau - \langle\hat{b}^\dagger\hat{b}\rangle_\tau^2\right) + \sin^2(\|\bar{\alpha}'\|)\cos^2(\|\bar{\alpha}'\|)\,\langle\hat{b}^\dagger\hat{b}\rangle_\tau \\
-\;& I_y\sin^2(\|\bar{\alpha}'\|)\cos^2(\|\bar{\alpha}'\|)\left[e^{-2i(\theta'-\theta_y)}\left(\langle(\hat{b}^\dagger)^2\rangle_\tau - \langle\hat{b}^\dagger\rangle_\tau^2\right) + e^{2i(\theta'-\theta_y)}\left(\langle\hat{b}^2\rangle_\tau - \langle\hat{b}\rangle_\tau^2\right)\right] \\
+\;& I_y\sin^2(\|\bar{\alpha}'\|)\cos^2(\|\bar{\alpha}'\|)\left(2\,\langle\hat{b}^\dagger\hat{b}\rangle_\tau + 1 - 2\,\langle\hat{b}^\dagger\rangle_\tau\,\langle\hat{b}\rangle_\tau\right) \\
+\;& i\sqrt{I_y}\,\sin(\|\bar{\alpha}'\|)\cos^3(\|\bar{\alpha}'\|)\left(e^{-i(\theta'-\theta_y)}\,\langle\hat{b}^\dagger\rangle_\tau - e^{i(\theta'-\theta_y)}\,\langle\hat{b}\rangle_\tau\right) \\
+\;& i\sqrt{I_y}\,\sin^3(\|\bar{\alpha}'\|)\cos(\|\bar{\alpha}'\|)\left[2e^{-i(\theta'-\theta_y)}\left(\langle(\hat{b}^\dagger)^2\hat{b}\rangle_\tau - \langle\hat{b}^\dagger\hat{b}\rangle_\tau\,\langle\hat{b}^\dagger\rangle_\tau + \frac{1}{2}\,\langle\hat{b}^\dagger\rangle_\tau\right)\right. \\
-\;& \left. 2e^{i(\theta'-\theta_y)}\left(\langle\hat{b}^\dagger\hat{b}^2\rangle_\tau - \langle\hat{b}^\dagger\hat{b}\rangle_\tau\,\langle\hat{b}\rangle_\tau + \frac{1}{2}\,\langle\hat{b}\rangle_\tau\right)\right].
\end{aligned} \tag{26}$$

The phononic correlation functions involving $\hat{b}$ and $\hat{b}^\dagger$ contribute with oscillations at frequency $\Omega$ while those involving $\hat{b}^2$ and $\hat{b}^{\dagger 2}$ give rise to $2\Omega$ oscillations. Collecting the corresponding coefficients one finds the following amplitude for the $2\Omega$ oscillating components:

$$|A_{2\Omega}(\tau)| = \frac{I_y(1+2n')}{8}\mathrm{e}^{-\lambda\tau}\sin^2(2\|\bar{\alpha}'\|)\sinh(2r)\ , \tag{27}$$

where the amplitude of the squeezing parameter $r = 2|c_2| = 2K|\mu_{\mathrm{s}}||\nu|^2$ is obtained from (14) and (10) by putting all pump amplitudes equal to $\nu$. Moreover we take $\lambda$ to comply with the observed oscillation time-scale and the time $\tau > 0$ such that $\lambda\tau \ll 1$.

In the last figure in the main text we have shown a fit of the experimental results for different pump intensities with the functional behaviour of $A_{2\Omega}$ predicted by the model in equation (27). We found an optimal value of the coupling parameter $\mu_{\mathrm{s}}$ for which the model agrees with the experiments. We used such a value for computing the amplitude $r$ of the squeezing parameter (defined in equation (10)) for all the experimental pump fluences. In particular $|\nu|^2$ is the number of photons per unit cell per pulse. We then computed the uncertainties in the position and momentum phonon operators as in equation (15). The results reported in the main text unveil photo-excited thermal squeezed vibrational states.

For completeness we also report the explicit time evolution of $\Delta^2_\tau \hat{N}_y$ in terms of both the amplitude $A_{2\Omega}$ of the $2\Omega$ frequency component and the amplitude $A_\Omega$ of the fundamental frequency component:

$$\Delta^2_\tau \hat{N}_y = A_0(\tau) + A_\Omega(\tau)e^{i\Omega\tau} + A^*_\Omega(\tau)e^{-i\Omega\tau} + A_{2\Omega}(\tau)e^{2i\Omega\tau} + A^*_{2\Omega}(\tau)e^{-2i\Omega\tau}, \tag{28}$$

where the explicit expression for $|A_\Omega(\tau)|$ is:

$$\begin{aligned} |A_\Omega(\tau)| &= \frac{1}{2}\sqrt{I_y}\,|z|\,e^{-\lambda\tau/2}\,\big|2e^{-\lambda\tau}\sin(2\|\bar{\alpha}'\|)\sin^2(\|\bar{\alpha}'\|)\,\big[(1+n'+n-(1+2n')\cosh(2r))\,e^{i\theta_r}\cosh(r) \\ &+ (n'-n+(1+2n')\cosh(2r))\,e^{2i\theta_z}\sinh(r)\big] \\ &- \sin(2\|\bar{\alpha}'\|)\left(1+2n\sin^2(\|\bar{\alpha}'\|)\right)\left(e^{i\theta_r}\cosh(r) - e^{2i\theta_z}\sinh(r)\right)\big|\,, \end{aligned} \tag{29}$$

where $|z|$ is the corresponding photo-exited displacement in the phonon and $\theta_z$ its phase.
We stress that, if the pump pulse does not generate squeezed phonons, vanishing squeezing parameter ($r \to 0$), the amplitudes of the two frequency components become,

$$\begin{aligned} |A_\Omega(\tau)| &= \frac{1}{2}\sqrt{I_y}\,z\,e^{-\lambda\tau/2}\,\left|\sin(2\|\bar{\alpha}'\|) + 2\sin(2\|\bar{\alpha}'\|)\sin^2(\|\bar{\alpha}'\|)\left(n-(n-n')e^{-\lambda\tau}\right)\right|\,, \\ |A_{2\Omega}(\tau)| &= 0, \end{aligned} \tag{30}$$

indicating the absence of the $2\Omega$ frequency component in the variance in absence of phonon squeezing.

From equations (29) and (27) one can notice that the damping constant $\lambda$, characterizing the dissipative phonon time evolution between the excitation and the probing process, contributes differently to $A_{2\Omega}$ and to $A_\Omega$, giving rise to different decay times for the two components and reproducing the experimental results.

## Supplemenary References


[1] Etchepare, J., Grillon, G., Chambaret, J. P., Hamoniaux, G. and Orszag, A. Polarization selectivity in time-resolved transient phase grating. *Optic. Comm.*, 63, 1987.

[2] Scott, J. F. and Porto, S. P. S. Longitudinal and transverse optical lattice vibrations in quartz. *Phys. Rev.*, 161, 1967.

[3] Umari, P., Pasquarello, A. and Dal Corso, A. Raman scattering intensities in $\alpha$-quartz: a first-principles investigation. *Phys. Rev. B*, 63:094305, 2001.

[4] Rundquist, A., Broman, J., Underwood, D. and Blank, D. Polarization-dependent detection of impulsive stimulated Raman scattering in $\alpha$-quartz. *J. Mod. Opt.*, 52, 2006.

[5] Mukamel, S. *Principles of Nonlinear Optical Spectroscopy.* Oxford University Press, 1995.

[6] Hussain, A. and Andrews, S. R. Absence of phase-dependent noise in time-domain reflectivity studies of impulsively excited phonons. *Phys. Rev. B*, 81:224304, 2010.

[7] Bachor, H. A. and Ralph, T. C. *A Guide to Experiments in Quantum Optics.* New York: Wiley, 2004.

[8] Titimbo, K. *Creation and detection of squeezed phonons in pump and probe experiments: a fully quantum treatment.* PhD thesis, University of Trieste, 2015.

[9] Dorner, B., Grimm, H. and Rzany, H. Phonon dispersion branches in $\alpha$ quartz. *J. Phys. C: Solid State Phys.*, 13:6607, 1980.

[10] Scott, J. F. Evidence of coupling between one- and two-phonon excitations in quartz. *Phys. Rev. Lett.*, 21:907–910, 1968.

[11] Scully, M. O. and Zubairy, M. S. *Quantum Optics.* Cambridge University Press, Cambridge UK, 1997.

[12] Alicki, R. and Lendi, K. *Quantum Dynamical Semigroups and Applications, Lect. Notes Phys. 717.* Springer-Verlag, Berlin, 2007.

[13] Breuer, H. P. and Petruccione, F. *The Theory of Open Quantum Systems.* Oxford University Press, New York, 2002.
